# Association of feeding patterns in infancy with later autism symptoms and neurodevelopment: a national multicentre survey

**DOI:** 10.1186/s12888-023-04667-2

**Published:** 2023-03-16

**Authors:** Xueli Xiang, Ting Yang, Jie Chen, Li Chen, Ying Dai, Jie Zhang, Ling Li, Feiyong Jia, Lijie Wu, Yan Hao, Xiaoyan Ke, Mingji Yi, Qi Hong, Jinjin Chen, Shuanfeng Fang, Yichao Wang, Qi Wang, Chunhua Jin, Tingyu Li

**Affiliations:** 1 Chongqing Key Laboratory of Childhood Nutrition and Health, Ministry of Education Key Laboratory of Child Development and Disorders, Children’s Nutrition Research Center, National Clinical Research Center for Child Health and Disorders, Children’s Hospital of Chongqing Medical University, Chongqing, China; 2 Xi’an Children’s Hospital, Xi’an, China; 3 Department of Children Rehabilitation, Hainan Women and Children’s Medical Center, Haikou, China; 4 Department of Developmental and Behavioral Pediatric, The First Hospital of Jilin University, Changchun, China; 5 Department of Children’s and Adolescent Health, Public Health College of Harbin Medical University, Harbin, China; 6 Department of Pediatrics, Tongji Hospital, Tongji Medical College, Huazhong University of Science and Technology, Wuhan, China; 7 Child Mental Health Research Center of Nanjing Brain Hospital, Nanjing, China; 8 Department of Child Health Care, The Affiliated Hospital of Qingdao University, Qingdao, China; 9 Maternal and Child Health Hospital of Baoan, Shenzhen, China; 10 Department of Child Healthcare, Shanghai Children’s Hospital, Shanghai Jiao Tong University, Shanghai, China; 11 Children’s Hospital Affiliated to Zhengzhou University, Zhengzhou, China; 12 NHC Key Laboratory of Birth Defect for Research and Prevention, Hunan Provincial Maternal and Child Health Care Hospital, Changsha, China; 13 Deyang Maternity & Child Healthcare Hospital, Deyang, China; 14 Department of Children Health Care, Capital Institute of Pediatrics, Beijing, China

**Keywords:** Autism spectrum disorder, Feeding patterns, Symptoms, Neurodevelopment, Multicenter survey

## Abstract

**Background:**

We aimed to compare differences in infant feeding patterns (breastfeeding and complementary food supplementation) between children with the autism spectrum disorder (ASD) and typically developing (TD) children through a multicentre study. The relationship between these patterns and later core symptoms and neurodevelopment in children with ASD was also investigated.

**Methods:**

We analysed breastfeeding and complementary feeding patterns in 1389 children with ASD and 1190 TD children. The Children Neuropsychological and Behavior Scale-Revision 2016 (CNBS-R2016) was used to assess neurodevelopmental levels. The Autism Behavior Checklist (ABC), Social Responsiveness Scale (SRS), Childhood Autism Rating Scale (CARS), and ASD Warning Behavior Subscale of the CNBS-R2016 were used to assess ASD symptoms.

**Results:**

Children with ASD had a shorter breastfeeding duration in infancy (8 (3–12) months vs. 10 (6–14) months, *P* < 0.001), later introduction of complementary foods (*P* < 0.001), and poorer acceptance of complementary foods (*P* < 0.001) than TD children. Total ABC and CARS scores were lower in the group of children with ASD who had been breastfed for 12 months or more than in the group who had been breastfed for less than 6 months. Children with ASD who were given complementary food after 6 months had lower general quotient (GQ), adaptive ability, fine motor and language scores than those who were given complementary food within 4–6 months. Children with ASD with poor acceptance of complementary foods had higher ABC and SRS scores and lower gross motor scores than those who had good acceptance.

**Conclusions:**

Children with ASD have a shorter duration of breastfeeding, a later introduction of complementary foods, and poorer acceptance of complementary foods than TD children. These feeding patterns may be related to the symptoms and growth of children with ASD. The research suggests that continued breastfeeding for longer than 12 months may be beneficial in reducing ASD symptoms and that infants who have difficulty introducing complementary foods should be followed up for neurodevelopment.

**Trial registration:**

The ethics committee of the Children’s Hospital of Chongqing Medical University approved the study. Approval Number: (2018) IRB (STUDY) NO. 121, and registered in the Chinese Clinical Trial Registry (Registration number: ChiCTR2000031194, registered on 23/03/2020).

**Supplementary Information:**

The online version contains supplementary material available at 10.1186/s12888-023-04667-2.

## Introduction

Autism spectrum disorder (ASD) is a neurodevelopmental condition marked by difficulty interacting with others, stereotypical behaviour, and narrow interests [1]. According to the Centers for Disease Control and Prevention (CDC), 1 in 44 children had autism in 2018, up approximately 24% from 2016 [2]. In China, the prevalence has reached 0.7% [3]. The situation with autism is dire, and the financial burden and psychological strain experienced by families of children with the condition is enormous. This makes it particularly important to look for possible protective factors and early manifestations of ASD.

ASD may be the result of genetic and environmental interactions. Numerous studies have demonstrated that early feeding patterns have a significant impact on the occurrence of ASD and its future progression [4–6]. Breastfeeding facilitates mother-infant bonding and neurodevelopment and cognitive development in children [7]. However, past research on the link between ASD and breastfeeding has not yielded the same results. Most studies have shown that breastfeeding may be protective against ASD [8–12] and is linked to reduced levels of autistic traits [5]. Studies have also revealed that prolonged breastfeeding may be associated with a reduced risk of ASD [13]. In contrast, there may be no link between breastfeeding and ASD, according to a US study that included 391 children with ASD in the analysis [13]. As growth and development mature, it becomes difficult to meet the nutritional needs of infants with breast milk or formula alone. Therefore, foods other than breast milk and formula need to be added, which are defined as complementary foods [14]. Many guidelines suggest adding after 6 months [14, 15]. However, the characteristics of children with ASD make it more difficult to introduce complementary foods compared to typically developing children [16]. Brzóska et al. [4] reported that compared to healthy children, children with ASD had more difficulty with the introduction of new foods and that solid and lumpy foods were introduced to them later. Another study found that children with ASD may develop food choice problems slowly after solid foods are introduced [17].

The majority of current studies focus on breastfeeding and the onset of ASD, and few studies have looked at feeding patterns and the progression of symptoms in later stages. To date, only a few studies on complementary feeding have been conducted in the West. Is there an association between breast milk and complementary feeding and ASD in Chinese children? Therefore, we conducted the first large-sample, multicentre study in China to investigate differences in several feeding patterns (e.g., breastfeeding duration, timing of complementary food introduction, and acceptance of complementary food) between children with ASD and typically developing (TD) children. The relationship of these patterns with the core symptoms and neurodevelopmental levels of children with ASD was simultaneously explored. We hypothesized that breastfeeding would be associated with fewer autistic traits in later childhood, whereas problematic eating behaviours in infants would be associated with higher autistic trait scores.

## Materials and methods

### Participants

This research is from the China Multi-Center Children Autism Project (CMCAP). The participants were recruited from thirteen cities in China between May 2018 and December 2019, including the Northern Region (Harbin, Qingdao, Changchun), the Southern Region (Haikou, Shenzhen, Changsha), the Eastern Region (Shanghai, Nanjing), the Western Region (Deyang, Chongqing, Xi'an) and the Central Region (Wuhan, Zhengzhou). We recruited children with ASD from special education institutions and outpatient clinics. Ultimately, 2762 children under 7 years old participated in this study, including 1457 children with ASD and 1305 TD children. In addition, 2579 children (1389 children with ASD and 1190 TD children) were ultimately included after 183 children who lacked all early feeding data were excluded.

### Selection criteria

The diagnosis of ASD was made by an experienced psychiatrist, psychologist, or neurodevelopmental paediatrician in conjunction with the DSM-5 and CARS. The exclusion criteria were as follows: (1) other neurodevelopmental and psychiatric disorders affecting growth or cognition, such as Rett's syndrome, epilepsy, cerebral palsy, and so on; (2) serious congenital disorders; and (3) acute and chronic infectious diseases in the last three months. The TD children were recruited from local schools and online volunteers. The exclusion criteria were as follows: 1) any serious congenital disorders; 2) any developmental disorders; and 3) acute and chronic infectious diseases in the last three months.

### Scales and questionnaires

#### General questionnaire

Caregivers of the participants completed a basic questionnaire including age, gender, residence, parental educational attainment, average annual household income, breastfeeding and complementary feeding during infancy. Caregivers were asked for information on feeding during infancy according to the World Health Organization (WHO) definition of breastfeeding. Content included the age at the beginning and end of months of exclusive breastfeeding (breast milk only or breast milk with water only), no breastfeeding (fully formula-fed) and partial breastfeeding (mixture of breast milk and formula) [8]. We used the duration of exclusive breastfeeding plus the duration of partial breastfeeding to calculate the duration of breastfeeding for the participating children, based on the completion of the questionnaire. The situation of complementary food included when complementary food was added and how well it was accepted. We asked parents to score their child's acceptance of complementary foods using a 3-point Likert scale. The answers were scored as 1 (poor), 2 (fair) and 3 (good).

#### Core symptoms and neurodevelopment scales

The Childhood Autism Rating Scale (CARS) is used by developmental paediatricians to assess autism disease severity and consists of 15 items. Each item is scored on a continuum from normal to severely abnormal. A score of 1 indicates a normal range for age, 2 indicates a mild abnormality, 3 indicates a moderate abnormality, and 4 indicates a severe abnormality. The total score ranges from 15 to 60, with scores of 30–36 indicating mild autism and 36 or more indicating severe autism [18].

The Autism Behavior Checklist (ABC) describes a range of typical autism-related behaviours. It is completed by the primary caregiver. The scale consists of 5 areas, sensory, relating, stereotypic behaviour, language, and social independence, with a cut-off score of 53 for ASD [19].

The Social Responsiveness Scale (SRS) measures social impairment in individuals with ASD [20]. The scale has 5 sections, including social awareness, cognition, communication, motivation, and autistic behaviour. A typically developing child should have a score < 65 [21, 22]. Higher scores indicate more severely impaired social functioning.

Professional doctors utilize the Children Neuropsychological and Behavior Scale-Revision 2016 (CNBS-R2016), established by the Capital Institute of Pediatrics, China, to evaluate children's neurodevelopment. The scale consists of six components (gross motor, fine motor, adaptive behaviour, language, personal social and communication warning behaviour). A total score or subscale developmental quotient (DQ) of < 70 indicates a developmental delay. A Communication Warning Behavior (CWB) subscale score of 12–30 indicates possible social impairment, while for a score > 30, ASD is highly suspected [23].

The above ABC, SRS, CARS, and CWB scales are all used for children with ASD.

### Statistical analysis

Statistical analysis was performed using SPSS 25.0 software. The distribution of each dataset for normality was verified using the Kolmogorov‒Smirnov goodness-of-fit test before analysis. We calculated the mean (SD) or median (IQR) for continuous variables and percentages for categorical variables. Mann‒Whitney tests and chi-square tests were used to analyse demographic factors. According to the duration of breastfeeding, we classified the variable breastfeeding duration (months) for both groups of children into: < 6 months; 6–12 months; and ≥ 12 months. Depending on the timing of the introduction of complementary foods, we divided the time of introduction of complementary foods (months) into three groups: ≤ 4 months of age; 4–6 months of age; > 6 months of age. When analysing the data among all the children, we found that no more than 7.3% were missing demographic data and no more than 8.3% were missing infant feeding data (breastfeeding duration: 8.3%, timing of complementary food introduction: 6.3% and acceptance of complementary food: 8.1%). To address the potential bias due to missing data, we performed multiple imputation by chained equations to generate five multiple imputation datasets. Furthermore, we analysed the link between feeding patterns during infancy and the occurrence of ASD using logistic regression. Age, gender, residence, annual family income and parental educational attainment varied between the ASD and TD groups. Therefore, we controlled for these variables as covariates in logistic regression analyses using the imputation data. Additionally, we performed a multiple linear regression corrected for the covariates described previously to investigate the relationship among feeding patterns, ASD symptoms, and developmental progress in childhood. *P* < 0.05 was considered statistically significant.

## Results

### Demographic characteristics

**Fig. 1:**
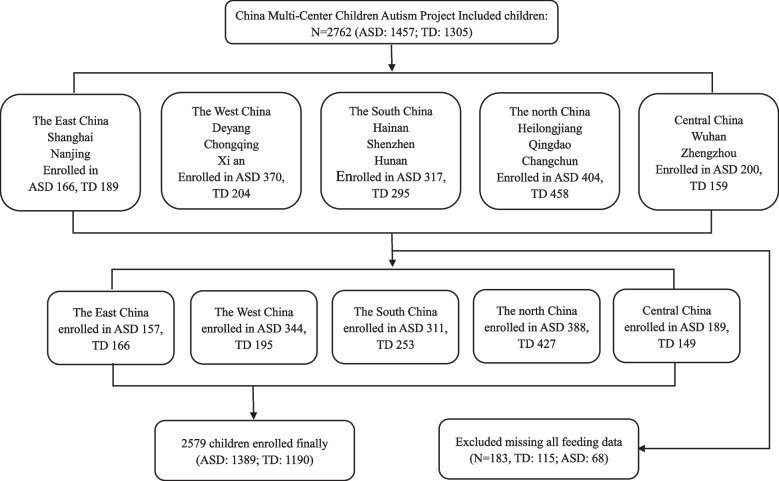
Flowchart of the study participants; ASD, autism spectrum disorder group; TD, typically developing group

This study enrolled 2762 children under 7 years old based on the inclusion and exclusion criteria. After excluding 183 children with missing data on all early feedings, 2579 children were ultimately included (Fig. 1). The ASD group included 1389 children, with 1138 males and 251 females and a median (IQR) age of 3.94 (3.11–4.90) years. The TD group included 1190 children, with 784 males and 406 females and a median (IQR) age of 4.40 (3.37–5.36) years. The two groups were significantly different in terms of age and gender (*P* < 0.05). Residence, annual family income, and parental education level between the two groups were also significantly different (Table 1). Therefore, we corrected for these demographic factors in a multivariate logistic regression analysis.

**Table 1:** Demographic characteristics of the participants in ASD and TD groups

Variable	ASD(*N* = 1389), Median (IQR)/N (%)	TD(*N* = 1190), Median (IQR)/N (%)	Z/χ2	*P*
**Age, years**	3.94(3.11,4.90)	4.40(3.37,5.36)	-6.101	** < 0.001**
**Gender**				
Male	1138(81.9)	784(65.9)	86.931	** < 0.001**
Female	251(18.1)	406(34.1)		
**Residence**				
Urban	1036(74.6)	1058(88.9)	90.230	** < 0.001**
Rural	334(24.0)	117(9.8)		
Miss	19(1.4)	15(1.3)		
**Annual family income, RMB**^a^				
< 50,000	615(44.3)	307(25.8)	146.726	** < 0.001**
50,000 ~ 100,000	403(29.0)	290(24.4)		
> 100,000	313(22.5)	506(42.5)		
Miss	58(4.2)	87(7.3)		
**Paternal education level**				
Primary education	22(1.6)	7(0.6)	48.743	** < 0.001**
Secondary education	553(39.8)	333(28.0)		
Secondary education above	798(57.5)	838(70.4)		
Miss	16(1.1)	12(1.0)		
**Maternal education level**				
Paternal education level	33(2.4)	14(1.2)	110.647	** < 0.001**
Primary education	623(44.9)	309(26.0)		
Secondary education	727(52.3)	863(72.5)		
Miss	6(0.4)	4(0.3)		

Data were shown as Median (IQR) or number (percentage)

Differences in annual family income and parental education levels were found between the ASD and TD groups after the multiple comparison for each subgroup, and passed the Bonferroni corrections

*ASD* autism spectrum disorder; *TD* typically developing, *IQR* inter-quartile range. Chi-square test and Mann–Whitney test were used in the analysis

^a^1 RMB≈0.155 US Dollars

### Comparison of infant feeding patterns between the TD and ASD groups

The infant feeding situations of the ASD and TD groups are summarized in Table 2. The results revealed that the children with ASD had a shorter breastfeeding duration in infancy than TD children (8 (3–12) months vs. 10 (6–14) months, *P* < 0.001). Also, compared to the TD group, the ASD group was introduced to complementary foods later (*P* < 0.001) and reported poorer acceptance of complementary foods per parent report (*P* < 0.001).

**Table 2:** Comparison of infant feeding between the TD and the ASD group

Variable	ASD, Median (IQR)/N (%)	TD, Median (IQR)/N (%)	Z	*P*
**Breastfeeding duration, months**	8(3,12)	10(6,14)	-4.905	** < 0.001**
**Breastfeeding duration, months**				
< 6 months	411(32.2)	231(21.3)	-4.901	** < 0.001**
≥ 6 months and less than 12 months	402(31.5)	396(36.4)		
≥ 12 months	465(36.4)	460(42.3)		
**Timing of complementary food**				
≤ 4 months	168(12.9)	168(15.1)	-4.697	** < 0.001**
> 4 months and ≤ 6 months	772(59.2)	745(66.9)		
> 6 months	363(27.9)	201(18.0)		
**Acceptance of complementary food**				
Good	721(56.8)	761(69.1)	-6.777	** < 0.001**
Fair	429(33.8)	301(27.3)		
Poor	120(9.5)	39(3.5)		

Mann–Whitney test was used in the analysis

*ASD* autism spectrum disorder, *TD* typically developing, *IQR* interquartile range

Table 3 compares the early feeding situation in the ASD and TD groups using logistic regression. In the crude model, there was no adjustment. Adjusted Model 1 considered the covariates of the child’s age, gender, residence, annual family income and parental educational attainment. To avoid interactions among the three infant feeding situations, we additionally corrected for the other two infant feeding situations. Multiple imputation was performed to address the presence of missing demographic and feeding data. The data after multiple imputation were used in adjusted Model 2 for the analysis, and the same covariates as those in Model 1 were adjusted. The results showed that children breastfed for 12 months or more had lower odds of having ASD than children less than 6 months, after adjusting for confounding factors (OR = 0.607, 95% CI: 0.486, 0.757, *P* < 0.001). Children with poor complementary food acceptance had higher odds of having ASD than children with good acceptance (OR = 2.672, 95% CI: 1.821, 3.921, *P* < 0.001).

**Table 3:** Comparison of infant feeding between the TD and the ASD children in different models

Variable	Crude Model ^a^		Adjusted Model 1^b^		Adjusted Model 2^c^	
	**OR (95%CI)**	***P***	**OR (95%CI)**	***P***	**OR (95%CI)**	***P***
**Breastfeeding duration, N1 = 2365, N2 = 2365, N3 = 2579**						
< 6 months	1[Reference]		1[Reference]		1[Reference]	
≥ 6 months and less than 12 months	0.571(0.461,0.706)	** < 0.001**	0.609(0.473,0.784)	** < 0.001**	0.625(0.493,0.792)	** < 0.001**
≥ 12 months	0.568(0.462,0.698)	** < 0.001**	0.599(0.469,0.764)	** < 0.001**	0.607(0.486,0.757)	** < 0.001**
**Timing of complementary food, N1 = 2417, N2 = 2417, N3 = 2579**						
> 4 months and ≤ 6 months	1[Reference]		1[Reference]		1[Reference]	
≤ 4 months	0.965(0.762,1.222)	0.768	1.028(0.771,1.372)	0.850	1.054(0.815,1.364)	0.689
> 6 months	1.743(1.428,2.128)	** < 0.001**	1.410(1.109,1.792)	**0.005**	1.451(1.167,1.805)	**0.001**
**Acceptance of complementary food, N1 = 2371, N2 = 2371, N3 = 2579**						
Good	1[Reference]		1[Reference]		1[Reference]	
Fair	1.504(1.258,1.799)	** < 0.001**	1.444(1.168,1.786)	**0.001**	1.403(1.157,1.701)	** < 0.001**
Poor	3.248(2.231,4.727)	** < 0.001**	3.140(2.005,4.917)	** < 0.001**	2.672(1.821,3.921)	** < 0.001**

*ASD* autism spectrum disorder, *TD* typically developing, *OR (95% CI)* odds ratio (95% confidence interval), *N1* the sample of Crude Model, *N2* the sample of Adjusted Model 1, *N3* the sample of Adjusted Model 2

^a^ Crude model: without any adjustment

^b^ Adjusted model 1: Multivariable logistic regression was used for adjusting for the child’s age, gender, residence, annual family income, paternal education level, maternal education level, and additionally adjusting for other two infant feeding situations

^c^ Adjusted model 2: On the basis of the original data, missing demographic data were imputed, as well as 214 data on breastfeeding, 162 data on time of introduction of complementary foods, and 208 data on reception of complementary food (both ASD and TD group). Adjusted for the same covariates in Model 1 with multiple imputed data

In addition, we compared the different feeding status in the first 6 months of life between the ASD and TD groups and found that children in the ASD group had lower rates of exclusive breastfeeding in the first 6 months than children in TD, and significantly higher rates of no breastfeeding than children in TD (all *P* < 0.001, Addition file 3). Multivariable logistic regression analysis found that partially and exclusively breastfed children were less likely to have ASD compared to children who were not breastfed (Addition file 4).

### Comparison of ABC, SRS, CARS, and CWB scores among autistic children with different early feeding patterns

On the basis of numerous imputed data, multivariate linear regression analyses were performed to investigate the effect of early feeding on core symptoms. Age, gender, residence, annual family income, paternal education level, maternal education level, and the two other feeding situations were adjusted as covariates (Table 4). The results revealed that the total ABC and CARS scores in children with ASD were lower in the group who were breastfed for 12 months or more than in the group who was breastfed for less than 6 months (β = -4.923, 95% CI: -8.214, -1.631, *P* < 0.01; β = -1.355, 95% CI: -2.330, -0.379, *P* < 0.01).

**Table 4:** Association between infant feeding and autism symptoms in children with ASD

Variable	Breastfeeding duration, β(95%CI)			Timing of complementary food, β(95%CI)				Acceptance of complementary food, β(95%CI)		
	< 6 months	6–12 months	≥ 12 months	4–6 months		≤ 4 months	> 6 months	Good	Fair	Poor
**ABC (*N* = 1328)**	Reference	-0.481 (-3.815,2.853)	-4.923^**^ **(-8.214, -1.631)**	Reference		0.932 (-3.163,5.027)	0.913 (-2.111,3.937)	Reference	4.229^**^ **(1.221,7.237)**	5.103^*^ **(0.288,9.919)**
**SRS (*N* = 1218)**										
Social awareness	Reference	-0.117 (-0.590, 0.355)	-0.147 (-0.616, 0.322)	Reference	-0.014 (-0.600, 0.572)		-0.330 (-0.767, 0.107)	Reference	0.464^*^ **(0.051, 0.877)**	1.101^**^ **(0.395, 1.806)**
Social cognition	Reference	-0.376 (-1.032, 0.279)	-0.628 (-1.278, 0.023)	Reference	0.020 (-0.781, 0.821)		-0.116 (-0.739, 0.506)	Reference	0.957^**^ **(0.355, 1.559)**	1.529^**^ **(0.585, 2.474)**
Social communication	Reference	0.236 (-1.077, 1.548)	-0.259 (-1.550, 1.033)	Reference	0.476 (-1.090, 2.042)		-0.326 (-1.572, 0.920)	Reference	1.935^**^ **(0.793, 3.078)**	3.455^***^ **(1.664, 5.246)**
Social motivation	Reference	0.037 (-0.691, 0.766)	-0.282 (-1.014, 0.450)	Reference	-0.096 (-0.982, 0.791)		-0.056 (-0.742, 0.630)	Reference	0.579 (-0.101, 1.260)	1.402^**^ **(0.353, 2.451)**
Autism behavior mannerisms	Reference	-0.027 (-1.935, 0.881)	-0.482 (-1.331, 0.367)	Reference	0.234 (-0.791, 1.259)		-0.300 (-1.091, 0.492)	Reference	1.255^**^ **(0.515, 1.995)**	2.052^**^ **(0.849, 3.255)**
SRS total scores	Reference	-0.290 (-3.625,3.046)	-1.891 (-5.173,1.390)	Reference	0.460 (-3.557,4.476)		-1.166 (-4.299,1.967)	Reference	5.268^**^ **(2.287,8.248)**	9.564^***^ **(4.934,14.194)**
**CARS (*N* = 1179)**	Reference	0.076 (-0.956,1.108)	-1.355^**^ **(-2.330, -0.379)**	Reference	1.174 (-0.088,2.435)		0.011 (-0.984,1.006)	Reference	-0.027 (-1.045,0.991)	0.476 (-0.897,1.849)
**Communication warning behavior (*N* = 962)**	Reference	0.198 (-3.293,3.689)	-2.071 (-5.460,1.318)	Reference	3.394 (-0.796,7.584)		0.524 (-2.725,3.772)	Reference	-0.109 (-3.166,2.949)	-1.866 (-6.519,2.786)

On the basis of the original data, missing demographic data were imputed, as well as 111 data on breastfeeding, 86 data on time of introduction of complementary foods, and 119 data on acceptance of complementary food (Only ASD group)

Multivariate linear regression was used for adjusting for the child’s age, gender, residence, annual family income, paternal education level, maternal education level, and additionally adjusting for other two infant feeding situations with multiple imputed data

*ASD* autism spectrum disorder, *β (95% CI)* regression coefficient (95% confidence interval)

^*^P < 0.05

^**^P < 0.01

^***^P < 0.001

Children with ASD with poor complementary food acceptance had higher total ABC and SRS scores than those with good acceptance (β = 5.103, 95% CI: 0.288, 9.919, *P* < 0.05; β = 9.564, 95% CI: 4.934, 14.194, *P* < 0.001). In addition, the former scored higher on the SRS awareness, cognition, communication, motivation and autism behaviour mannerisms subscales (*P* < 0.01). Similarly, children with fair complementary food acceptance also had higher total ABC and SRS scores than those with good acceptance (β = 4.229, 95% CI: 1.221, 7.237, *P* < 0.01; β = 5.268, 95% CI: 2.287, 8.248, *P* < 0.01). The former had higher scores on the awareness, cognition, communication and autism behaviour mannerisms subscales (*P* < 0.05). The effect of infant feeding on ASD symptoms was similar before and after the imputation of missing data (Additional file 1). The scale scores that were inconsistent before and after imputation all had p values close to 0.05. However, we did not find an association between breastfeeding and complementary feeding and CWB scores.

### Comparison of neurodevelopmental levels among autistic children with different early feeding patterns

As shown in Table 5, after adjusting for covariates, children with ASD who were given complementary food after 6 months of age had lower general quotient scores (GQ, β = -3.584, 95% CI: -6.370, -0.799, *P* = 0.012) and fine motor (β = -3.275, 95% CI: -6.324, -0.226, *P* = 0.035), adaptive ability (β = -4.023, 95% CI: -7.119, -0.927, *P* = 0.011) and language ability DQ scores (β = -4.684, 95% CI: -8.575, -0.794, *P* = 0.018) than those who were given complementary food within 4–6 months. There were no statistically significant differences in the DQ of each subscale in children with ASD who had supplemental foods introduced within 4 months or 4–6 months of age. Children with ASD who had poor acceptance of complementary foods had a lower gross motor DQ score than those who had good acceptance of complementary foods (β = -5.204, 95% CI: -9.603, -0.804, *P* = 0.020). The effect of infant feeding on the CNBS-R 2016 developmental quotient score was similar before and after the imputation of missing data (Additional file 2). However, we did not find an association between breastfeeding or non-breastfeeding in the first 6 months of life and autism symptoms (Additional file 5).

**Table 5:** Association between infant feeding and CNBS-R 2016 developmental quotient in children with ASD

Variable	GQ		Gross motor		Fine motor		Adaptive behavior		Language		Personal-social	
	**β(95%CI)**	***P***	**β(95%CI)**	***P***	**β(95%CI)**	***P***	**β(95%CI)**	***P***	**β(95%CI)**	***P***	**β(95%CI)**	***P***
**Breastfeeding duration, *N* = 975**												
< 6 months	Reference		Reference		Reference		Reference		Reference		Reference	
6–12 months	-1.252 (-4.178,1.673)	0.401	1.007 (-2.346,4.359)	0.556	-1.097 (-4.419,2.225)	0.516	-3.208 (-6.433, 0.017)	0.051	-2.144 (-6.124,1.835)	0.291	-0.826 (-4.153,2.502)	0.627
≥ 12 months	0.969 (-1.961,3.898)	0.516	1.458 (-1.968,4.885)	0.401	1.301 (-1.930,4.533)	0.428	0.120 (-3.217,3.457)	0.943	0.826 (-3.129,4.781)	0.682	1.391 (-1.717,4.498)	0.380
**Timing of complementary food, *N* = 975**												
4–6 months	Reference		Reference		Reference		Reference		Reference		Reference	
≤ 4 months	-1.966 (-5.541, 1.609)	0.281	-2.462 (-6.411, 1.487)	0.222	-2.431 (-6.365, 1.503)	0.225	-1.421 (-5.440, 2.597)	0.488	-2.509 (-7.324, 2.305)	0.307	-1.157 (-5.182,2.868)	0.573
> 6 months	-3.584 (-6.370, -0.799)	**0.012**	-2.915 (-5.966, 0.135)	0.061	-3.275 (-6.324, -0.226)	**0.035**	-4.023 (-7.119, -0.927)	**0.011**	-4.684 (-8.575, -0.794)	**0.018**	-2.768 (-5.836,0.299)	0.077
**Acceptance of complementary food, *N* = 975**												
Good	Reference		Reference		Reference		Reference		Reference		Reference	
Fair	1.034 (-1.609,3.677)	0.442	1.267 (-1.648,4.183)	0.394	1.046 (-1.722,3.815)	0.458	1.737 (-1.279,4.753)	0.258	1.107 (-2.444,4.658)	0.541	0.123 (-2.949,3.195)	0.937
Poor	-3.143 (-7.136, 0.850)	0.123	-5.204 (-9.603, -0.804)	**0.020**	-0.494 (-4.882,3.895)	0.825	-3.164 (-7.635,1.306)	0.165	-2.821 (-8.249,2.606)	0.308	-4.155 (-8.663, 0.354)	0.071

On the basis of the original data, missing demographic data were imputed, as well as 111 data on breastfeeding, 86 data on time of introduction of complementary foods, and 119 data on acceptance of complementary food (Only ASD group)

Multivariate linear regression was used for adjusting for the child’s age, gender, residence, annual family income, paternal education level, maternal education level, and additionally adjusting for other two infant feeding situations with multiple imputed data

*ASD* autism spectrum disorder, *β (95% CI)* regression coefficient (95% confidence interval)

## Discussion

Early feeding is crucial to a child’s growth [24, 25] and has attracted much attention in ASD. Previous studies have shown that early feeding affects children's neurodevelopment and later-life healthy eating habits [26, 27]. Therefore, it is critical to identify these factors early. However, few studies have investigated whether early feeding patterns and later symptoms in children with ASD are related [5, 28]. Therefore, we conducted this first large-scale national study including 13 collaborating centres to further explore whether this linkage exists.

This study analysed early-life breastfeeding duration, the timing of the introduction of complementary foods, and the acceptance of complementary foods to investigate their relationship with neurodevelopmental levels and core symptoms of autism. The results showed that the breastfeeding duration was shorter in the ASD group than in the control group. This association remained significant after adjusting for sociodemographic factors. This is in line with other research that suggested that prolonged nursing periods could lower the chance of children developing ASD [12, 28–32]. After comparing 673 children with ASD and 876 control children, Soke et al. [29] proposed that mothers of children with ASD reported a shorter duration of breastfeeding.

It is difficult to distinguish between ASD outcomes due to insufficient breastfeeding and breastfeeding difficulties due to ASD symptoms. The former may be a result of infants with ASD receiving insufficient breast milk early on, which prevents them from receiving the full benefits of breastfeeding. These benefits include benign neurological stimulation, more consistent parent‒child interaction, and more nutrition [9, 33, 34]. The latter may be associated with sensory disturbances in children with ASD. Breastfeeding provides richer sensory and tactile stimuli, thereby affecting the breastfeeding process of infants with ASD and sensory processing disorders [5].

In addition, most children in both groups had supplemental foods introduced within 4–6 months of age, but the ASD group had later introductions and poorer acceptance than the TD group. Similar to our findings, Brzoska et al. [4] found that when comparing normal children and children with autism, the latter were introduced to solid foods later and bottle-fed for longer periods. The results suggest that there may be a link between the delayed introduction of complementary foods and autism.

Most guidelines advocate for the introduction of supplemental foods after 6 months [14, 15], due to the declining nutritional value of breast milk to fulfil growing infants’ needs [35]. But children with ASD have sensory abnormalities, have difficulty accepting change and are fussy about food, which may contribute to the delayed introduction and poor acceptance of complementary foods [16]. Therefore, infants who have particular difficulty in adding complementary foods should be followed up over time to exclude development delays. However, the evidence for this explanation is still unclear, and further study is needed to confirm whether difficulties with supplemental feeding in young children may be used as an early predictor of ASD.

We also linked breastfeeding and complementary feeding to ASD core symptoms. The ABC, CARS and SRS were used in the study; the former two were used to assess autism symptoms [18, 19], and the latter was used to rate social dysfunction [21]. The total ABC and CARS scores were lower in the group of children with ASD who had been breastfed for 12 months or more than in the group who had been breastfed for less than 6 months. This further illustrates the relevance of breastfeeding to children's neurodevelopment. A previous study showed that prolonged breastfeeding may help children's cognitive development [36]. A multicentre prospective birth cohort of Spanish children also demonstrated this benefit in children with ASD. This study found that a longer breastfeeding duration improved cognitive function and reduced autistic traits and that these benefits persisted beyond 12 months of breastfeeding [28]. Another study used the SRS to rate the autistic trait scores of breastfed and formula-fed children with autism at 2 months and found that the latter had higher scores [5]. This is also consistent with our findings that breastfeeding may be beneficial in reducing ASD symptoms. A recent clinical trial found that repetitive behaviours in ASD may be improved by oxytocin [37]. The breastfeeding process is an important driver of oxytocin secretion, which may be a reason why it reduces ASD symptoms. Some have also found that parent‒child interactions can influence the severity of ASD [38] and that a mother's warm emotions may be beneficial for symptoms [39]. In our study, we also considered the possibility that increased mother-infant interaction during breastfeeding might be associated with less severe ASD symptoms later in life.

As mentioned above, many studies have shown that breastfeeding has positive effects on children's neuropsychological development that may extend into adulthood [40, 41]. However, other studies have failed to conclude that breastfeeding is beneficial to children's intellectual development after controlling for various confounding factors [42]. Our study found that breastfeeding duration was not significantly associated with neurodevelopment in children with autism. This may be related to the older age of the children when we observed their developmental levels.

Another finding was that children with ASD who had complementary foods introduced after 6 months of age had more severe developmental delays in adaptive, fine motor, language, and general developmental levels than those who had complementary foods introduced within 4–6 months of age. This may be because the severity of the neurodevelopmental delay affects the timing of the introduction of complementary foods in infants. Therefore, children with severe developmental delays have a later introduction of complementary foods. Studies have found that infants who had complementary foods introduced before 26 weeks of age have better exercise capacity than infants who had complementary foods introduced after 26 weeks of age [43]. However, the relationship between supplemental food introduction and neurodevelopment may be influenced by body weight and oral development, so further cohort studies controlling for relevant confounders are needed to clarify the relationship.

Children with ASD often exhibit behaviours such as food refusal, a narrow food spectrum, and stereotypical eating [6]. Difficulty with the introduction of complementary foods may be an early sign of feeding difficulties in children with ASD. To date, no clear conclusions have been drawn about the relationship between feeding difficulties and ASD symptoms and developmental levels. Some studies have shown that feeding difficulties are positively related to ASD severity. Patton et al. [44] reported that children with higher levels of ASD severity had more difficulty accepting unfamiliar foods. According to additional studies, feeding difficulties in children with ASD are linked to core symptoms. Picky eaters have more severe restrictive repetitive behaviours [6]. Findings from Zachor and Benltzchak et al. [45] showed that children who are picky eaters are less adaptable and have more impairments in daily living, social skills and motor development. Our study found that children with poor acceptance of complementary foods had higher total ABC and SRS scores and lower gross motor scores than children with ASD who had good acceptance. This indicated that children with ASD who had poor acceptance of complementary foods had more severe symptoms and poorer developmental levels, confirming previous research.

The main strength of this study is that it investigated breastfeeding and complementary feeding patterns in Chinese children with ASD at a multicentre level. This study provides a preliminary view that breastfeeding continued for 12 months and beyond may be beneficial for children with ASD. Previous research has revealed that feeding difficulties may be one of the earliest signs of ASD. Therefore, children who are at risk for difficulty with food introduction and receiving complementary foods should be screened and treated early.

Our study also has some limitations. First, this was a cross-sectional investigation and any causal relationship between early feeding and the occurrence of ASD cannot be explained. Second, this was a retrospective study where breastfeeding and complementary feeding were conducted by asking primary caregivers, and recall bias exists. Moreover, the ASD group was not drawn at random but rather through special education settings and outpatient clinics, which reduced the representativeness of the sample. Third, because the mothers' reasons for discontinuing breastfeeding and the specifics of introducing complementary foods were not examined, we cannot rule out the possibility that they had an impact on the outcome. Therefore, further studies are needed.

## Conclusion

In summary, our study shows that children with ASD were breastfed for a shorter period of time, had complementary foods introduced later, and had poor acceptance of complementary foods compared to TD children. Breastfeeding and the introduction of complementary foods may be associated with core symptoms and neurodevelopment in children with ASD in later life. This study also suggests that breastfeeding for 12 months and beyond may be beneficial to the neurodevelopment of children with ASD. Children who are breastfed for shorter periods and have difficulty introducing complementary foods should be carefully assessed for neurodevelopmental disorders. And we need to further investigate the interrelationship between early feeding and ASD.

## Supplementary Information

**Additional file 1.** 

**Additional file 2.** 

**Additional file 3.** 

**Additional file 4.** 

**Additional file 5.**

## Data Availability

The datasets generated during the current study are not publicly available due to ethical restrictions but are available from the corresponding author on reasonable request.

## Abbreviations

ABC: Autism Behavior Checklist
ASD: Autism Spectrum Disorder
CARS: Childhood Autism Rating Scale
CDC: Centers for Disease Control and Prevention
CMCAP: China Multi-Center Children Autism Project
CNBS-R2016: Children Neuropsychological and Behavior Scale-Revision 2016
CWB: Communication Warning Behavior
DQ: Developmental quotient
DSM-5: Diagnostic and Statistical Manual of Mental Disorders, fifth edition
GQ: General quotient
IQR: Inter-quartile range
OR (95% CI): Odds ratio (95% confidence interval)
SD: Standard Deviation
SRS: Social Responsiveness Scale
TD: Typically-developing
WHO: World Health Organization
β: Regression coefficient
