# Supplementary material for: Association of feeding patterns in infancy with later autism symptoms and neurodevelopment: a national multicentre survey
Source: BMC Psychiatry. 2023 Mar 16;23:174. doi: 10.1186/s12888-023-04667-2 (PMC10022051; doi:10.1186/s12888-023-04667-2)
Supplement: Supplementary file 1 — Additional file 1. [file 12888_2023_4667_MOESM1_ESM.docx]

**Table S1.** Effect of infant feeding on autism symptoms in ASD children (original data)

| **Variable** | **Breastfeeding duration, β(95%CI)** | | | **Timing of complementary food, β(95%CI)** | | | | **Acceptance of complementary food, β(95%CI)** | | |
| --- | --- | --- | --- | --- | --- | --- | --- | --- | --- | --- |
|  | <6 months | 6-12 months | ≥12 months | 4-6 months | | ≤4 months | >6 months | Good | Fair | Poor |
| **ABC (N=1328)** | Reference | -0.309  (-3.906,3.287) | **-5.981****  **(-9.431, -2.530)** | Reference | 0.098  (-4.381,4.576) | | 0.048  (-3.280,3.375) | Reference | **4.257****  **(1.128,7.385)** | 3.780  (-1.150,8.709) |
| **SRS (N=1218)** |  |  |  |  |  | |  |  |  |  |
| Social awareness | Reference | 0.064  (-0.466, 0.593) | -0.200  (-0.715, 0.315) | Reference | 0.052  (-0.597, 0.701) | | -0.442  (-0.937, 0.053) | Reference | **0.573***  **(0.111, 1.035)** | **1.279****  **(0.524, 2.033)** |
| Social cognition | Reference | -0.426  (-1.173, 0.321) | **-0.933***  **(-1.660, -0.206)** | Reference | 0.177  (-0.738, 1.092) | | -0.457  (-1.154, 0.241) | Reference | **1.014****  **(0.363, 1.665)** | **1.588****  **(0.525, 2.651)** |
| Social communication | Reference | 0.591  (-0.867, 2.050) | -0.266  (-1.685, 1.153) | Reference | -0.150  (-1.938, 1.638) | | -0.791  (-2.154, 0.572) | Reference | **2.322*****  **(1.050, 3.593)** | **2.970****  **(0.893, 5.046)** |
| Social motivation | Reference | 0.088  (-0.724, 0.901) | -0.465  (-1.255, 0.325) | Reference | -0.380  (-1.375, 0.615) | | -0.285  (-1.044, 0.473) | Reference | 0.620  (-0.088, 1.328) | **1.355***  **(0.198, 2.512)** |
| Autism behavior mannerisms | Reference | 0.183  (-0.758, 1.124) | -0.656  (-1.571, 0.260) | Reference | 0.071  (-1.082, 1.224) | | -0.523  (-1.403, 0.356) | Reference | **1.340****  **(0.520, 2.161)** | **1.674***  **(0.334, 3.014)** |
| SRS total scores | Reference | 0.476  (-3.258,4.209) | -2.609  (-6.239,1.021) | Reference | -0.422  (-4.984,4.140) | | -2.522  (-6.011,0.967) | Reference | **5.938*****  **(2.683,9.192)** | **8.900****  **(3.583,14.216)** |
| **CARS (N=1179)** | Reference | 0.255  (-0.886,1.396) | **-1.710****  **(-2.806, -0.614)** | Reference | **1.437***  **(0.010,2.864)** | | 0.020  (-1.026,1.066) | Reference | -0.146  (-1.144,0.853) | 0.574  (-0.970,2.117) |
| **Communication warning**  **behavior (N=962)** | Reference | 1.324  (-2.547,5.196) | -2.304  (-6.028,1.420) | Reference | 1.951  (-2.867,6.769) | | 0.766  (-2.810,4.342) | Reference | -0.456  (-3.851,2.940) | -2.582  (-7.699,2.535) |

*Multivariate linear regression was used for adjusted for child’s age, gender, residence, annual family income, paternal education level, maternal education level and additionally adjusted for other two infant feeding situations with original data.*

*ASD=autism spectrum disorder; β (95% CI) =regression coefficient (95% confidence interval). *P<0.05, **P<0.01, ***P<0.001.*
