# Supplementary material for: Association of feeding patterns in infancy with later autism symptoms and neurodevelopment: a national multicentre survey
Source: BMC Psychiatry. 2023 Mar 16;23:174. doi: 10.1186/s12888-023-04667-2 (PMC10022051; doi:10.1186/s12888-023-04667-2)
Supplement: Supplementary file 2 — Additional file 2. [file 12888_2023_4667_MOESM2_ESM.docx]

**Table S2.** Effect of infant feeding on CNBS-R 2016 developmental quotient in ASD children (original data)

| **Variable** | **GQ** | | **Gross motor** | | **Fine motor** | | **Adaptive behavior** | | **Language** | | **Personal-social** | |
| --- | --- | --- | --- | --- | --- | --- | --- | --- | --- | --- | --- | --- |
|  | **β(95%CI)** | ***P*** | **β(95%CI)** | ***P*** | **β(95%CI)** | ***P*** | **β(95%CI)** | ***P*** | **β(95%CI)** | ***P*** | **β(95%CI)** | ***P*** |
| **Breastfeeding duration** |  |  |  |  |  |  |  |  |  |  |  |  |
| <6 months | Reference |  | Reference |  | Reference |  | Reference |  | Reference |  | Reference |  |
| 6-12 months | -1.552  (-4.886,1.782) | 0.361 | 0.948  (-2.729,4.624) | 0.613 | -1.192  (-4.809,2.426) | 0.518 | -3.657  (-7.342, 0.029) | 0.052 | -2.667  (-7.191,1.857) | 0.248 | -1.173  (-4.927,2.580) | 0.540 |
| ≥12 months | 0.927  (-2.273,4.127) | 0.570 | 1.499  (-2.028,5.026) | 0.404 | 1.941  (-1.529,5.412) | 0.272 | -0.120  (-3.655,3.416) | 0.947 | 0.674  (-3.666,5.014) | 0.761 | 1.019  (-2.582,4.621) | 0.579 |
| **Timing of complementary food** |  |  |  |  |  |  |  |  |  |  |  |  |
| 4-6 months | Reference |  | Reference |  | Reference |  | Reference |  | Reference |  | Reference |  |
| ≤4 months | -0.884  (-5.043, 3.275) | 0.677 | -2.489  (-7.057, 2.079) | 0.285 | -1.480  (-5.981, 3.020) | 0.519 | 0.004  (-4.588, 4.596) | 0.988 | -0.630  (-6.258, 4.999) | 0.826 | -0.133  (-4.800,4.534) | 0.955 |
| >6 months | -4.107  (-7.170, -1.045) | **0.009** | -3.106  (-6.483, 0.271) | 0.071 | -3.908  (-7.236, -0.581) | **0.021** | -4.422  (-7.816, -1.027) | **0.011** | -5.613  (-9.774, -1.452) | **0.008** | -3.327  (-6.777,0.123) | 0.059 |
| **Acceptance of complementary food** |  |  |  |  |  |  |  |  |  |  |  |  |
| Good | Reference |  | Reference |  | Reference |  | Reference |  | Reference |  | Reference |  |
| Fair | 1.815  (-1.086,4.717) | 0.220 | 1.846  (-1.348,5.040) | 0.257 | 1.508  (-1.650,4.665) | 0.349 | 2.561  (-0.653,5.776) | 0.118 | 2.561  (-1.384,6.507) | 0.203 | 0.622  (-2.650,3.893) | 0.709 |
| Poor | -3.344  (-7.748,1.060) | 0.136 | -5.303  (-10.152, -0.454) | **0.032** | -1.029  (-5.822,3.765) | 0.674 | -3.152  (-8.033,1.728) | 0.205 | -2.601  (-8.591,3.388) | 0.394 | -4.786  (-9.753,0.181) | 0.059 |

*Multivariate linear regression was used for adjusted for child’s age, gender, residence, annual family income, paternal education level, maternal education level and additionally adjusted for other two infant feeding situations with original data.*

*ASD=autism spectrum disorder; β (95% CI) =regression coefficient (95% confidence interval).*
