# Supplementary figures and images for: Association of feeding patterns in infancy with later autism symptoms and neurodevelopment: a national multicentre survey
Source: BMC Psychiatry. 2023 Mar 16;23:174. doi: 10.1186/s12888-023-04667-2 (PMC10022051; doi:10.1186/s12888-023-04667-2)

**Figure S1** Breastfeeding status under the first six months in TD and ASD group


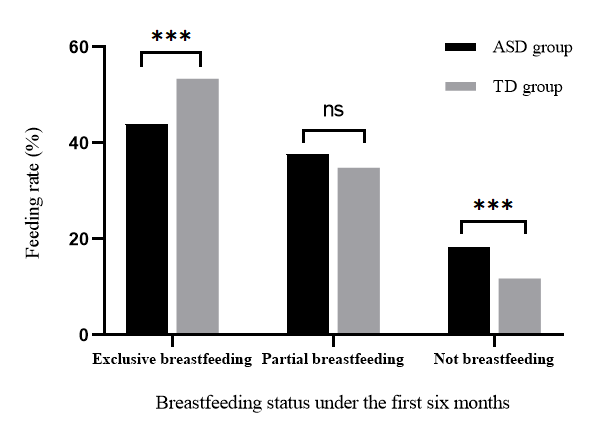

Supplement: Supplementary file 3 — Additional file 3. [file 12888_2023_4667_MOESM3_ESM.docx]
