# Supplementary material for: Association of feeding patterns in infancy with later autism symptoms and neurodevelopment: a national multicentre survey
Source: BMC Psychiatry. 2023 Mar 16;23:174. doi: 10.1186/s12888-023-04667-2 (PMC10022051; doi:10.1186/s12888-023-04667-2)
Supplement: Supplementary file 4 — Additional file 4. [file 12888_2023_4667_MOESM4_ESM.docx]

| **Variable** | **N** | **Crude Model ^a^** | | **Adjusted Model 1^b^** | |
| --- | --- | --- | --- | --- | --- |
|  |  | **OR (95%CI)** | ***P*** | **OR (95%CI)** | ***P*** |
| Not breastfeeding | 362 | 1[Reference] |  | 1[Reference] |  |
| Partial breastfeeding | 861 | 0.696(0.540, 0.897) | **0.005** | 0.746(0.561, 0.993) | **0.044** |
| Exclusive breastfeeding | 1142 | 0.530(0.415, 0.677) | **＜0.001** | 0.551(0.418,0.727) | **＜0.001** |

**Table S3** Comparison of feeding status for the first six months between the TD and ASD group

*^a^ Crude model: without any adjustment.*

*^b^ Adjusted model 1: Multivariable logistic regression was used for adjusting for the child’s age, gender, residence, annual family income, paternal education level, and maternal education level.*

*ASD=autism spectrum disorder; TD=typically developing;* *OR (95% CI) =* *odds ratio (95% confidence interval).*
