# Supplementary material for: Association of feeding patterns in infancy with later autism symptoms and neurodevelopment: a national multicentre survey
Source: BMC Psychiatry. 2023 Mar 16;23:174. doi: 10.1186/s12888-023-04667-2 (PMC10022051; doi:10.1186/s12888-023-04667-2)
Supplement: Supplementary file 5 — Additional file 5. [file 12888_2023_4667_MOESM5_ESM.docx]

**Table S4** Association between feeding status for the first six months and autism symptoms and developmental level in ASD children

| **Variable** | **Not breastfeeding β(95%CI)** | **Partial breastfeeding** | | **Exclusive breastfeeding** | |
| --- | --- | --- | --- | --- | --- |
|  |  | **β(95%CI)** | ***P*** | **β(95%CI)** | ***P*** |
| **ABC (N=1226)** | Reference | 2.766  (-1.195,6.726) | 0.171 | -2.831  (-6.711, 1.049) | 0.153 |
| **SRS (N=1131)** | Reference | 2.227  (-1.810,6.263) | 0.279 | -0.829  (-4.800, 3.142) | 0.682 |
| **CARS (N=1090)** | Reference | 0.625  (-0.602, 1.852) | 0.318 | -0.339  (-1.539, 0.860) | 0.579 |
| **Communication warning**  **behavior (N=879)** | Reference | 1.860  (-2.272, 5.993) | 0.377 | 0.523  (-3.504, 4.551) | 0.799 |
| **GQ (N=890)** | Reference | 0.288  (-3.243, 3.820) | 0.873 | 0.025  (-3.414, 3.464) | 0.989 |
| **Gross motor (N=890)** | Reference | 2.397  (-1.454, 6.248) | 0.222 | 1.392  (-2.360, 5.144) | 0.467 |
| **Fine motor (N=890)** | Reference | 0.009  (-3.813, 3.830) | 0.997 | 0.582  (-3.141, 4.306) | 0.759 |
| **Adaptive behavior (N=890)** | Reference | 0.231  (-3.698,4.160) | 0.908 | -1.421  (-5.249,2.407) | 0.466 |
| **Language (N=890)** | Reference | -1.152  (-5.960,3.655) | 0.638 | -0.678  (-5.362, 4.006) | 0.776 |
| **Personal-social (N=890)** | Reference | -0.095  (-4.065,3.875) | 0.962 | 0.322  (-3.546,4.190) | 0.870 |

*Multivariate linear regression was used for adjusting for the child’s age, gender, residence, annual family income, paternal education level, and maternal education level.*

*ASD=autism spectrum disorder; β (95% CI) =regression coefficient (95% confidence interval).*
